# Sustained Activation of CLR/RAMP Receptors by Gel-Forming Agonists

**DOI:** 10.3390/ijms232113408

**Published:** 2022-11-02

**Authors:** Chia Lin Chang, Zheqing Cai, Sheau Yu Teddy Hsu

**Affiliations:** 1 Department of Obstetrics and Gynecology, Chang Gung Memorial Hospital Linkou Medical Center, Chang Gung University, Kweishan, Taoyuan 20878, Taiwan; 2 CL Laboratory LLC, Gaithersburg, MD 20878, USA; 3 Adepthera LLC, San Jose, CA 95138, USA

**Keywords:** adrenomedullin, CLR/RAMP receptor, RAMP2, vasodilation, endothelium, liquid gel, treatment-resistant hypertension

## Abstract

**Background**: Adrenomedullin (ADM), adrenomedullin 2 (ADM2), and CGRP family peptides are important regulators of vascular vasotone and integrity, neurotransmission, and fetoplacental development. These peptides signal through CLR/RAMP1, 2, and 3 receptors, and protect against endothelial dysfunction in disease models. As such, CLR/RAMP receptor agonists are considered important therapeutic candidates for various diseases. **Methods and Results**: Based on the screening of a series of palmitoylated chimeric ADM/ADM2 analogs, we demonstrated a combination of lipidation and accommodating motifs at the hinge region of select peptides is important for gaining an enhanced receptor-activation activity and improved stimulatory effects on the proliferation and survival of human lymphatic endothelial cells when compared to wild-type peptides. In addition, by serendipity, we found that select palmitoylated analogs self-assemble to form liquid gels, and subcutaneous administration of an analog gel led to the sustained presence of the peptide in the circulation for >2 days. Consistently, subcutaneous injection of the analog gel significantly reduced the blood pressure in SHR rats and increased vasodilation in the hindlimbs of adult rats for days. **Conclusions**: Together, these data suggest gel-forming adrenomedullin analogs may represent promising candidates for the treatment of various life-threatening endothelial dysfunction-associated diseases such as treatment-resistant hypertension and preeclampsia, which are in urgent need of an effective drug.

## 1. Introduction

The calcitonin/CGRP family peptides include calcitonin, amylin, calcitonin gene-related peptides (α-CGRP and β-CGRP), adrenomedullin (ADM), and adrenomedullin 2 (ADM2 or intermedin [IMD]) [1,2]. The closely related ADM, ADM2, and CGRPs signal through receptor complexes consisting of calcitonin receptor-like receptor (CLR) and one of the three receptor activity-modifying proteins (RAMP1, 2, and 3) [1,3,4,5,6]. Although CGRPs mainly act through CLR/RAMP1, ADM has a high affinity for CLR/RAMP2 and 3 [1,2]. On the other hand, ADM2 is a mild nonselective agonist for the three CLR/RAMP receptors.

ADM, ADM2, and CGRPs play important roles in the regulation of vasotone, cardiovascular morphogenesis, neurotransmission, pain perception, and fetoplacental development [7,8,9,10,11,12,13,14,15]. Among them, ADM is essential for normal blood and lymphatic vessel development as well as the maintenance of vessel integrity [8,16,17,18,19,20,21,22,23,24,25,26], whereas ADM2 is important for regulating vascular lumen enlargement [27]. On the other hand, CGRPs are crucial regulators of sensory neurotransmission and migraine pain [28,29,30,31,32,33,34]. In addition, the CLR/RAMP3 system has been shown to be essential for regulating normal lymphatic vessel functions [35]. Because these peptides exhibit potent cardioprotective, neuroprotective, and renoprotective effects in a variety of disease models [24,25,36,37,38,39], CLR/RAMP receptors are considered valid targets for ameliorating endothelial dysfunction-associated diseases such as treatment-resistant hypertension (RHTN), preeclampsia, and secondary lymphedema [40]. However, due to the lack of stable analogs, the utility of CLR/RAMP receptor agonists for disease treatment remains to be vetted.

We have recently reported that the receptor-activation activity of several palmitoylated chimeric ADM/ADM2 analogs is one order greater than that of wild-type ADM and ADM2 [41]. Based on that observation, we screened a series of ADM and ADM/ADM2 analogs to determine whether the enhanced bioactivity of these analogs is a result of palmitoylation modification and/or select motifs in the chimeras.

Screening of a series of ADM/ADM2 analogs showed that palmitoylation and a proper junctional sequence are important for acquiring enhanced bioactivity and that the putative hinge region of these peptides is amenable to modification. By serendipity, we also discovered that select palmitoylated ADM and ADM/ADM2 analogs self-assemble to form a liquid gel in situ. Subcutaneous injection of an ADM analog gel solution in rats led to the sustained presence of the peptide in the circulation for days. Administration of the analog gel also resulted in sustained reduction of blood pressure in spontaneous hypertensive SHR rats and prolonged localized vasodilation in rat hindlimbs. Together, these data suggest that the gel-forming analogs represent promising candidates for the treatment of severe hypertensive disorders such as treatment-resistant hypertension (RHTN) and preeclampsia as well as for localized treatment of traumatic wounds, secondary lymphedema, or myocardial infarction while avoiding potential systemic effects in the acute or long-term treatment regimes.

## 2. Results

### 2.1. Select Palmitoylated ADM/ADM2 Analogs Potently Activate CLR/RAMP1 and 2 Receptors

**Figure 1:**
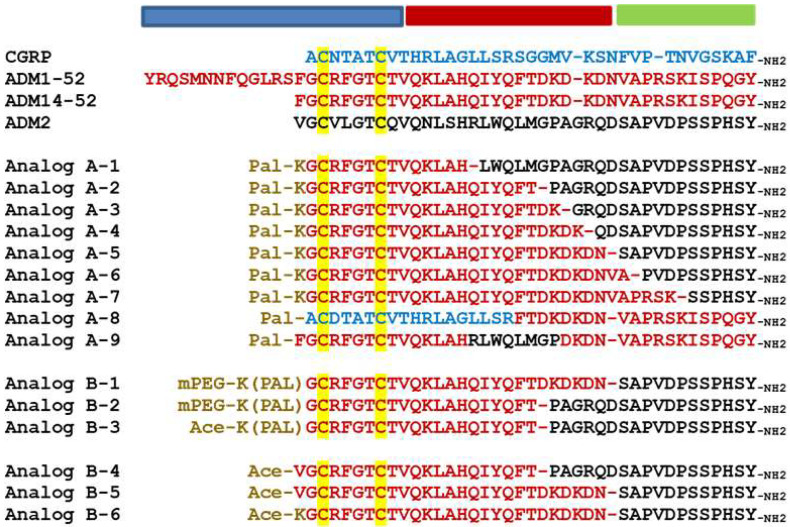
**Alignment of ADM family peptides and chimeric analogs.** The alignment includes wild-type peptides and Analogs A-1 to A9 and Analogs B-1 to B-6. In chimeric analog sequences, residues derived from CGRP, ADM, and ADM2 are indicated by blue, red, and black letters, respectively. The disulfide ring-forming cysteines are indicated by a yellow background. The N-terminal modifications, including lysine-conjugated palmitoylation (Pal-K or K(pal)) and mini-PEGylation (abbreviated as mPEG), are indicated by brown letters. Dash lines are added in select sequences to improve alignment. Acetylation is abbreviated as Ace. The putative activation, binding, and junctional regions of these peptides are indicated by blue, green, and red horizontal bars above the alignment, respectively.

In a recent study, we reported that palmitoylated ADM and chimeric ADM/ADM2 analogs exhibit enhanced receptor-activation activity toward CLR/RAMP1 and/or 2, and the EC_50_ values of the most potent chimeric analog for CLR/RAMP1 and 2 were >one order lower than that of wild-type peptides (e.g., Analog A-5 in Figure 1 and Table 1) [41]. To study the attributes that allow these analogs to gain an enhanced receptor-activation activity, we screened the activities of a series of chimeric analogs that contain a 14- to 32-residue N-terminal ADM fragment and a C-terminal ADM2 fragment (Figure 1, Analogs A-1 to A-7). Like the prototypic Analog A-5, these peptides were palmitoylated at the N-terminus.

The agonistic activity is described as EC_50_ and the maximum activity in the percentage of positive control. The positive control for CLR/RAMP1 is β-CGRP, and the control for CLR/RAMP2 and 3 is ADM1-52. All EC_50_ values were presented as nM. Additional controls included ADM14-52 and ADM2. EC_50_ values which are < or = 1 nM are indicated by bold letters. In these assays, multiple positive controls showed similar results in at least three different assays, and all compounds were first dissolved in DMSO to avoid solubility issue-associated variations. Analogs that have enhanced activity for CLR/RAMP1 and 2 are indicated by a yellow background. Analogs that exhibit enhanced activity for CLR/RAMP2 (i.e., with a 5-fold reduction of EC_50_ when compared to wild-type ADM) are indicated by a blue background.

Receptor-activation assays showed that CGRP has an EC_50_ of 1.8–2.9 nM for CLR/RAMP1. The EC_50_ values of wild-type ADM (i.e., ADM1-52 or ADM14-52) and ADM2 for CLR/RAMP1 are >100 nM. On the other hand, the EC_50_ values of ADM and ADM2 for CLR/RAMP2 were 9–12 nM and 70 nM, respectively. By contrast, the EC_50_ values of Analogs A-2 to A-6 for CLR/RAMP1 and 2 were 0.2–0.8 nM and 0.6–1.0 nM, respectively (Table 1). Although the maximum activity of these analogs for CLR/RAMP1 was ~42–58%, their maximum activity for CLR/RAMP2 was 110–130%.

By contrast, the Analog A-7, which has a 32-residue ADM fragment, had potencies similar to that of wild-type ADM. The EC_50_ values of Analog A-1, which has a 14-residue ADM fragment, were >1000 nM for CLR/RAMP1 and 2. Likewise, analogs that contained an ADM-ADM2-ADM or a CGRP-ADM chimeric sequence had low potencies (i.e., Analogs A-8 and A-9, Table 1). Thus, only peptides with select combinations of chimeric sequences gained enhanced receptor-activation activity.

### 2.2. N-Terminal Palmitoylation Is Important for Enhancing Chimeric Analogs’ Receptor-Activation Activity

To study whether palmitoylation itself is important for enhancing bioactivity, we screened variants that contained different types of N-terminal modification. These variants included analogs with palmitoylation and a mini-PEG modification (Analogs B-1 and B-2, Table 1) or a palmitoylation at the epsilon position of the N-terminal lysine residue (Analog B-3) as well as those lacked an N-terminal modification (Analogs B-4 to B-6). Although Analogs B-1, 2, and 3 had EC_50_ similar to those of parental palmitoylated analogs, nonpalmitoylated Analogs B-4, 5, and 6 had potencies inferior or similar to those of wild-type peptides (Table 1), indicating that an N-terminal palmitoylation is important for enhancing the receptor-activation activity, and a hydrophilic moiety such as mini-PEG does not affect this characteristic.

**Figure 2:**
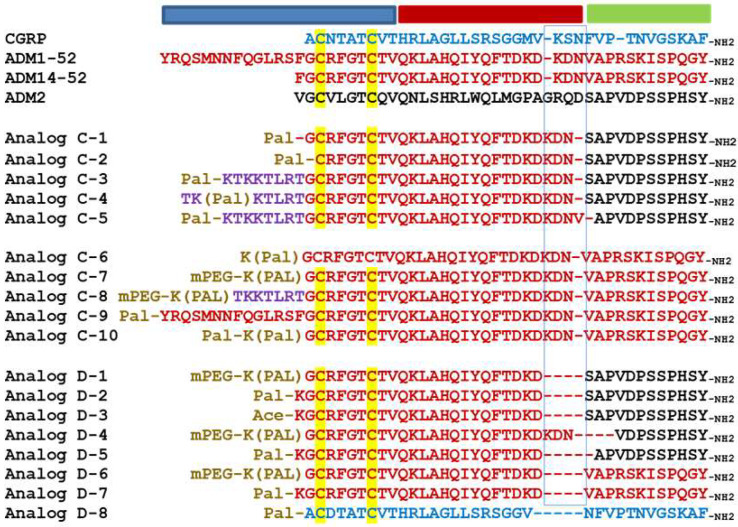
**Alignment of analogs with an N-terminal sequence modification or a truncation at the junctional region.** The alignment includes CGRP (blue letters), adrenomedullin (red letters), and adrenomedullin 2 (black letters) as well as Analogs C-1 to C-9 and Analogs D-1 to D-8. Cysteine residues that form a disulfide ring are indicated by a yellow background. A putative collagen-binding motif is indicated by purple letters. The origin of individual residues in chimeric analogs is indicated by text colors. The N-terminal modifications, including lysine-conjugated palmitoylation (Pal-K or K(pal)) and mini-PEGylation (abbreviated as mPEG), are indicated by brown letters. Sequence gaps are indicated by dash lines. The putative activation, binding, and junctional regions of these analogs are indicated by blue, green, and red horizontal bars above the alignment, respectively. The motif corresponding to the hinge region of CGRP is indicated by a blue rectangle box.

To study whether sequence complexity at the N-terminal activation domain affects the palmitoylation-enhanced bioactivity, we examined additional Analog A-5 variants that contain residue deletion (Analogs C-1 and 2) or a putative collagen-binding motif at the N-terminus (i.e., the TKKTLRT motif in Analogs C-3, 4, and 5; Figure 2) [42]. This analysis showed, except for Analogs C-2, all other analogs have enhanced receptor-activation activities. The deletion of residues N-terminal to the first cysteine in Analog C-2 rendered the analog a CLR/RAMP1-selective analog, indicating that a suitable motif N-terminal to the first cysteine is important for enhancing the bioactivity.

In addition, we studied the bioactivity of ADM analogs with different N-terminal modifications (Analogs C-6, 7, 8, 9, and 10, Figure 2). Although Analogs C-6, 7, 8, and 9, which contained a palmitoylation or a palmitoylation plus a mini-PEG modification, exhibited a >3-fold increase of potency toward CLR/RAMP2, the Analog C-10 which contained two palmitic acid modifications had reduced activity toward CLR/RAMP2.

### 2.3. Palmitoylated Chimeric and ADM Analogs with a 3-Residue Truncation at the Putative Hinge Region Retained the Enhanced Receptor-Activation Activity

Because chimeric analogs with different junctional sequences (i.e., Analogs A-2, 3, 4, 5, and 6) exhibit enhanced bioactivity, we then hypothesized that the motif at the junctional region could be amenable to structural changes. As such, we studied additional chimeric variants with a deletion of 3 or 4 residues at the junctional region as well as ADM analogs with similar truncations (Analogs D-1 to D-7, Figure 2). Although analogs with a 3-residue deletion at the C-terminus of the ADM fragment (Analogs D-1 and 2) retained the enhanced bioactivity, nonpalmitoylated Analog D-3 had activity similar to that of wild-type ADM. On the other hand, the analog with a 3-residue deletion of the C-terminal ADM2 fragment (Analog D-4) had a reduced activity when compared to Analog D-1 and 2. A deletion of 4 residues in Analog D-5 drastically reduced the ability to activate CLR/RAMP1 and 2.

Likewise, palmitoylated ADM analogs with a 3-residue deletion at the corresponding hinge region (Analogs D-6 and D-7) retained the enhanced receptor-activation characteristics similar to that of Analogs C-6 and C-7. By contrast, a palmitoylated CGRP analog with the same truncation (Analog D-8) had minimal bioactivity.

To determine whether the activity-enhancing modifications also improve the receptor-activation activity toward CLR/RAMP3, we analyzed the CLR/RAMP3-activation activity of select analogs (i.e., Analogs B-1, C-7, D-1, D-2, D-6, and D-7, Table 1). Although the EC_50_ of wild-type ADM for CLR/RAMP3 was ~0.4 nM, Analogs B-1 and D-1 had an EC_50_ of 0.06–0.07 nM. Likewise, Analogs C-7, D-2, D-6, and D-7 had EC_50_ values in the subnanomolar range. These data suggested that select analogs such as Analogs B-1 and D-1 have enhanced activity toward all three CLR/RAMP receptors.

### 2.4. Analogs with Enhanced Activity Potently Stimulated the Proliferation and Survival of Primary Human Lymphatic Microvascular Endothelial (HLME) Cells

**Figure 3:**
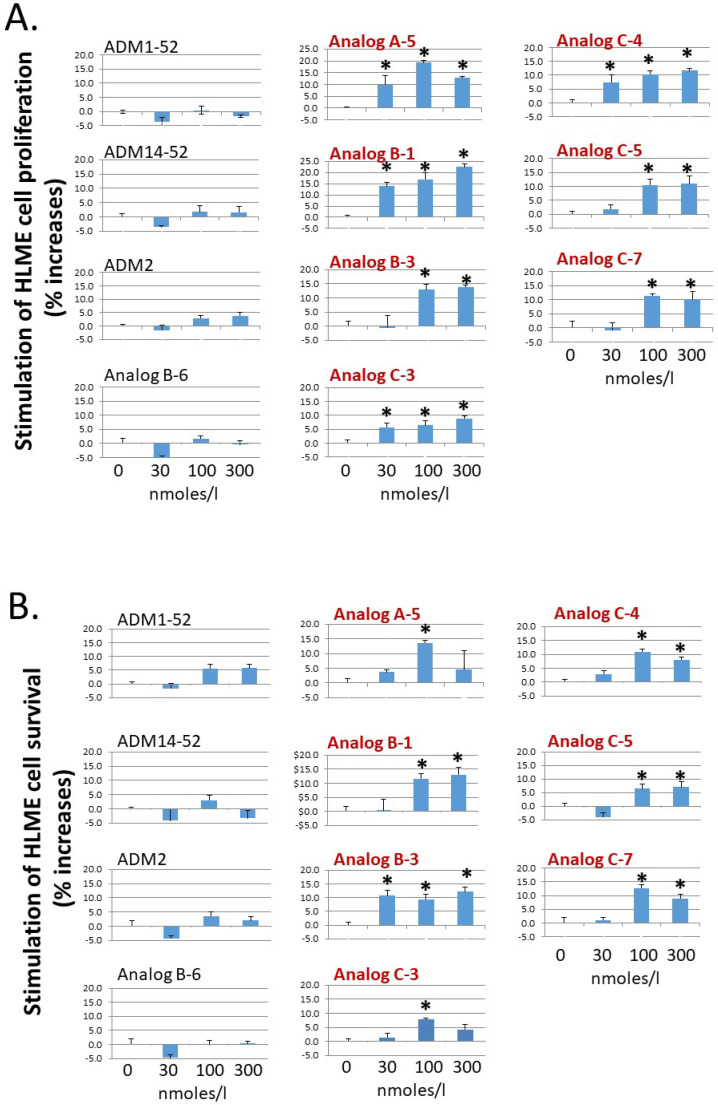
Analogs with enhanced receptor-activation activity potently stimulate the proliferation and survival of primary HLME cells. Analogs A-5, B-1, B-3, C-3, C-4, C-5, and C-7 significantly increased (**A**) the proliferation of primary human lymphatic microvascular endothelial (HLME) cells in a medium with 2% FBS and (**B**) the survival of HLME cells in medium with 0.1% FBS. HLME cells were cultured for 72 h, and cell viability was quantified with the MTT assay. The wild-type peptides (ADM1-52, ADM14-52, and ADM2) and a nonlipidated analog (Analog B-6) had negligible effects on HLME cell proliferation and survival. Analogs with an enhanced CLR/RAMP2-activation activity are indicated by red letters. Data are mean ± SEM of quintuplicate samples. Similar results were obtained in at least three different experiments. *, Significantly different from controls.

Because ADM-CLR/RAMP2 signaling is a key regulator of endothelial and lymphendothelial functions, analogs with enhanced CLR/RAMP2-activation activity could have improved bioactivity toward basic functions of vascular endothelial cells. As such, we studied the effect of select analogs, which have an enhanced CLR/RAMP2-activation activity (i.e., Analogs A-5, B-1, B-3, C-3, C-4, C-5, and C-7), on the proliferation and survival of primary human lymphatic microvascular endothelial (HLME) cells in vitro. Consistent with the hypothesis, these analogs dose-dependently stimulated HLME cell proliferation and survival at the 30–100 nmoles/L range (Figure 3A,B). On the other hand, wild-type ADM (ADM1-52 and ADM14-52), ADM2, and a nonpalmitoylated chimeric peptide (Analog B-6) had minimal effects on HLME cell proliferation and survival at the same doses. In addition, select analogs such as Analogs A-5, C-3, C-4, and C-7 appeared to have a greater stimulatory effect on proliferation and/or survival at the 100 nmoles/L level when compared to the 300 nmoles/L group. These data may indicate that a high pharmacological dose of these analogs may have a deleterious effect on cell metabolism and cell cycle progression or lead to the down-regulation of CLR/RAMP receptors.

### 2.5. Select Palmitoylated Analogs Self-Assembled to Form Gels In Situ and Gel Formation Slowed the Passage of Analog C-6 Molecules through the Centricon Filter

By serendipity, we also found that select palmitoylated ADM and chimeric analogs such as Analogs B-1, C-2, C-6, and D-1 form liquid gels in situ at 10–20% levels. On the other hand, the closely related peptides such as Analogs C-1 and C-7 did not form a gel at the same concentrations. Because self-assembled peptide gel has been used to deliver the somatostatin analog lanreotide for the treatment of acromegaly and neuroendocrine tumors (NETs) [43,44,45,46], we hypothesized that the gel-forming analogs may have improved pharmacological characteristics. Because Analog C-6 is one of the best-characterized analogs we had studied, we used it as a prototype to study the characteristics of gel-forming analogs.

**Figure 4:**
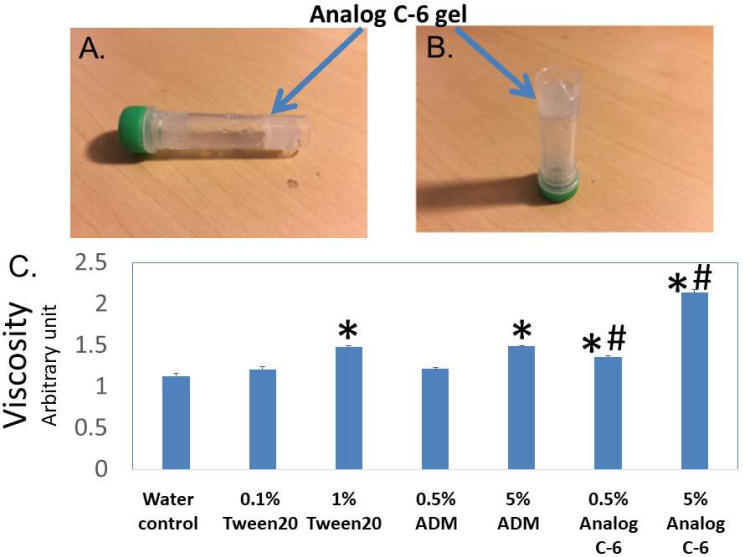
**Analog C-6 self-assembles to form gels in situ, and the analog solution has a high viscosity**. (**A**,**B**) Representative pictures of 11% Analog C-6 gel solution at 30 min after dissolution. The gel mass is indicated by blue arrows. (**C**) The relative viscosity of a 5% glucose solution (water solution), 1% Tween 20 solution, 0.5% and 5% wild-type ADM solutions in 5% glucose, and 0.5% and 5% Analog C-6 solutions in 5% glucose. The relative viscosity was measured by a Rheosense viscometer *, Significantly different from the water control. #, Significantly different from the wild-type ADM solution with the same peptide level (i.e., 0.5% or 5%).

Visual analysis showed that Analog C-6 solution exhibits gel-like characteristics at the 6–20% level within 20–30 min, and this transformation limited the mobility of the analog solution (Figure 4A,B). After gel formation, the solution lost the free-moving aqueous fluid characteristics. Consistent with the visual analysis, studies of the viscosity using a viscometer showed that the viscosities of a 1% Tween 20 solution, 5% ADM solution, 0.5% Analog C-6 solution, and 5% Analog C-6 solution are significantly higher than that of water. Although the viscosity of wild-type ADM and Analog C-6 solutions increased in a dose-dependent manner, the viscosity of Analog C-6 solution was significantly higher than that of wild-type ADM at the same concentration (Figure 4C). These data suggest that the Analog C-6 has a high propensity to form gels.

**Figure 5:**
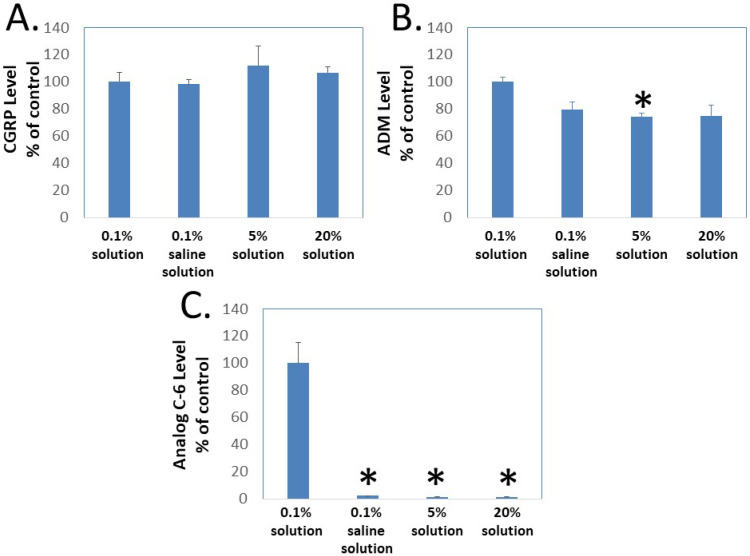
**Gel formation hinders the passage of Analog C-6 molecules through Centricon membrane filters.** To determine whether gel formation reduces the peptide’s freedom to move, we analyzed the passage of (**A**) CGRP, (**B**) ADM, and (**C**) Analog C-6 molecules through the Centricon membrane filter (MW cutoff: 30,000). The testing solutions included 0.1% peptide in 5% glucose (0.1% solution), 0.1% peptide in saline solution as well as 5% and 20% peptide in 5% glucose (5% and 20% solution). For 5% and 20% solution, one-milligram aliquots of the peptides were first dissolved at the target concentration and diluted to the 0.1% level 10 min later. Aliquots of these solutions were then dispensed into individual Centricon columns before centrifugation for 15 min (2000× *g*). Levels of peptides in the elute were determined by specific CGRP or ADM EIA. *, Significantly different from the 0.1% solution control group. Data are mean ± SEM of triplicate samples.

To determine whether gel formation restrains the movement of Analog C-6 molecules, we studied the ability of CGRP, wild-type ADM, and Analog C-6 molecules to pass through the Centricon filter at different concentrations. In this analysis, aliquots of peptides were first dissolved at target concentrations (i.e., 0.1%, 5%, and 20%), and the high-concentration samples were then diluted to the 0.1% level 10 min later before the centrifugation separation step. Analysis of peptide levels in the elutes of Centricon columns showed that the levels of CGRP in elutes from the 0.1% solution in saline, and the 0.1%, 5%, and 20% solution in 5% glucose are similar (Figure 5A). Although the levels of ADM in the elute from the 0.1% solution in saline and the 0.1% solution in 5% glucose are similar, the level of ADM in the elute from the 5% and 20% solution groups was lower than that of the 0.1% solution group and the difference was significant between the 0.1% and 5% solution groups (Figure 5B). By contrast, the Analog C-6 appeared to have a low solubility in the saline solution and formed liquid gel at 5% and 20% levels. Only a trace amount of Analog C-6 monomer went through the membrane, and the level of Analog C-6 in the elute from the 0.1% solution was 30–50 folds higher than those from the 0.1% in saline, 5% solution, and 20% solution groups (Figure 5C). These data suggested that most Analog C-6 molecules in 5% and 20% solutions were sequestered in the gel and cannot pass through the Centricon filter membrane.

### 2.6. Subcutaneous Administration of Analog C-6 Gel Solution Led to the Sustained Presence of the Peptide in the Circulation of Rats

**Figure 6:**
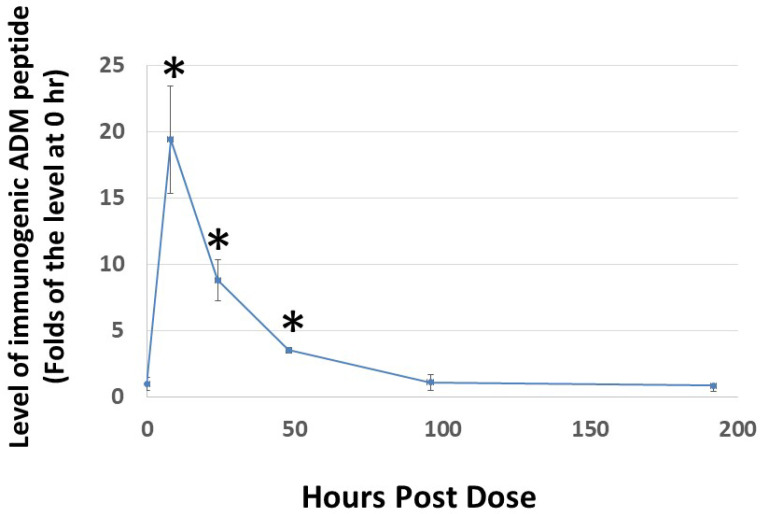
**Subcutaneous administration of Analog C-6 gel solution leads to the sustained presence of the peptide in the circulation of rats.** The circulating level of Analog C-6 was significantly increased in the circulation from 8 to 48 h after injection. An aliquot of Analog C-6 gel solution (32 mg in 16% solution) was delivered subcutaneously in male adult Sprague–Dawley rats, and blood samples were collected at pre-dose, 8, 24, 48, 96, and 192 h after injection. The peptide level was determined by specific ADM EIA. *, Significantly different from controls at 0 h. Data are mean ± SEM of three separate animals.

Because the rate of dissociation of hydrogels depends on electrostatic interactions among the monomeric peptide, solvent, and solutes, the release of Analog C-6 from the gel solution is likely affected by various environmental factors [47,48,49]. Therefore, it is important to characterize the release kinetics of the analog gel in vivo before we can reasonably design studies to investigate its efficacy in animals. Analysis of the level of Analog C-6 in rats after subcutaneous injection of a 16% gel solution showed that the level of Analog C-6 peaks at 8 h after injection, and the peptide level remained significantly elevated at 2 days after administration (Figure 6). These data suggested that the self-assembled gel formulation allows the Analog C6 peptide to be slowly released in vivo given that wild-type ADM is known to have a short half-life of 20–30 min.

### 2.7. Analog C-6 Gel Solution Has a Prolonged Effect on Hemodynamics in SHR Rats and Vascular Blood Flow in Rat Hindlimbs

**Figure 7:**
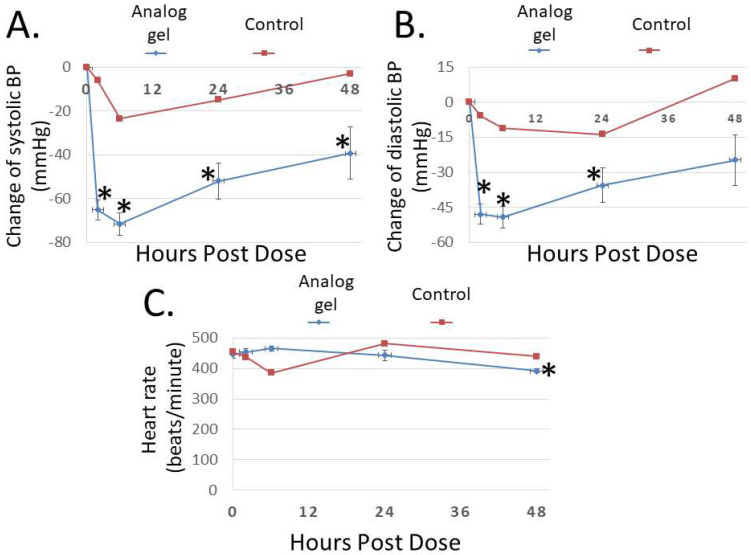
**The Analog C-6 gel solution has a prolonged effect on blood pressure in SHR rats.** Subcutaneous injection of Analog C-6 gel solution led to the significant reduction of (**A**) systolic blood pressure at 2, 6, 24, and 48 h after administration and (**B**) diastolic blood pressure at 2, 6, and 24 h after administration in male SHR rats (8%; 8 mg/100 µL injection). On the other hand, Analog C-6 gel injection only affected (**C**) the heart rate 48 h after injection. The hemodynamics was recorded on 0, 2, 6, 24, and 48 h after drug administration. Data are mean ± SEM of five separate animals. In addition, the change of hemodynamics of a representative control animal, which received saline injection, is included for comparison (red lines) *, Significantly different from the measurement at 0 h.

To study the bioactivity of Analog C-6 gel solution in vivo, we first investigated its effect on blood pressure in SHR rats. As shown in Figure 7A,B, subcutaneous injection of the gel solution (8%; 8 mg/100 µL solution) led to a prolonged reduction of systolic and diastolic pressures, and the systolic blood pressure remained significantly reduced at 48 h after administration. On the other hand, the injection of saline had a comparatively small effect on blood pressure in control animals. Unlike wild-type ADM and ADM2 which are known to induce tachycardia after administration [3], Analog C-6 gel solution had a minimal acute effect on the heart rate of animals (Figure 7C). However, a significant reduction in the heart rate was recorded at 48 h after injection of the gel solution.

**Figure 8:**
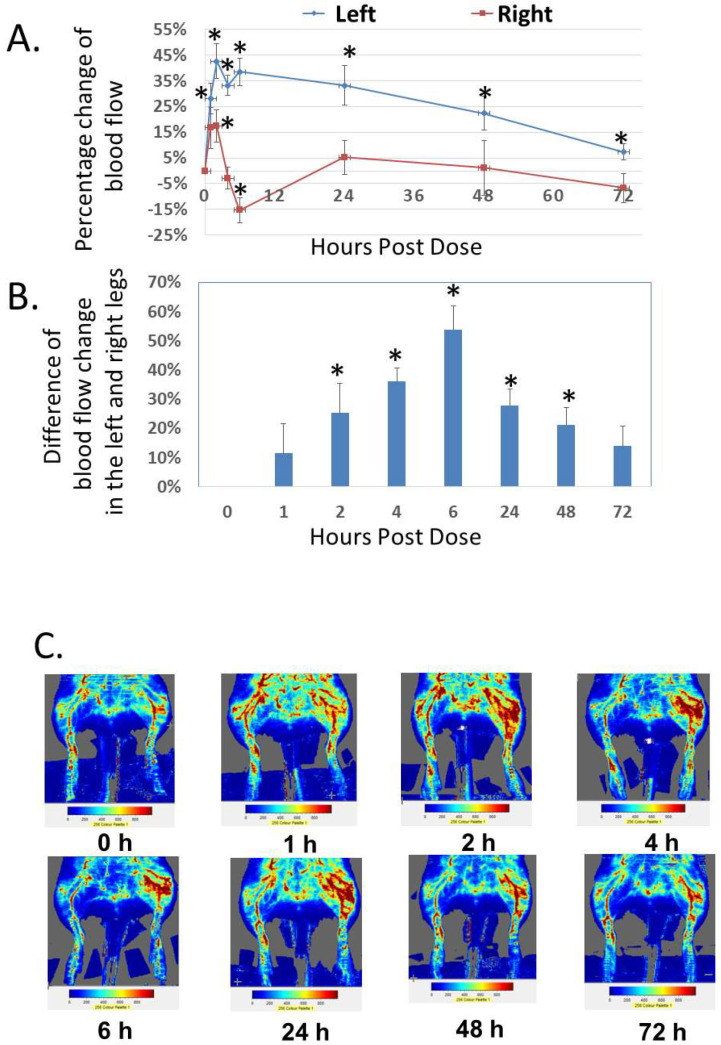
**Analog C-6 gel solution has a prolonged effect on dermal blood flow in the hindlimbs of Sprague–Dawley rats.** (**A**) Percentage changes of dermal blood flow in the left and right hindlimbs at 0, 1, 2, 4, 6, 24, 48, and 72 h after a subcutaneous injection of gel solution (8 mg in 8% solution) in the left hindlimb of anesthetized adult male Sprague–Dawley rats. The rats received the injection after the basal scans with a Doppler imager at the beginning of the experiment. The blood flow in the hindlimbs was again scanned at 1, 2, 4, 6, 24, 48, and 72 h after the start of the experiment. Data are reported as percent change from the average of baseline scans and are mean ± SEM of six separate animals. *, Significantly different from the basal level at 0 h. (**B**) The difference of changes in blood flow between the left (treatment side) and right (control side) hindlimbs at 0, 1, 2, 4, 6, 24, 48, and 72 h after the injection of gel solution at the left hindlimb. *, significantly different between the two hindlimbs. (**C**) Representative scans of dermal blood flow at different time points in animals that received an Analog C-6 gel solution injection.

In addition to the analysis of systemic effect, we studied the local effect of Analog C-6 gel solution on dermal blood flow in the hindlimbs of adult rats (Figure 8A–C). Subcutaneous injection of Analog C-6 gel solution (8%; 8 mg/100 µL solution) in the left hindlimb (i.e., treatment side) led to a sustained and significant increase of dermal blood flow in the left hindlimb at 1–72 h after injection. Although Analog C-6 gel administration also increased dermal blood flow in the right hindlimb (control side) at 2 and 6 h after drug administration, this increase disappeared 24 h after injection. The increase in dermal blood flow within the left hindlimb was significantly higher than that of the right hindlimb at 2, 4, 6, 24, and 48 h after administration, suggesting the gel solution has a prolonged local effect on vasodilation (Figure 8B).

## 3. Discussion

Studies of ADM and ADM/ADM2 analogs showed that select palmitoylated analogs have (1) EC_50_ values 3- to 100-fold lower than those of wild-type peptides and (2) the ability to form liquid gels in situ. The self-assembled gel formulation allows the peptide to be slowly released in vivo and exert a lasting effect on hemodynamics and dermal blood flow. The analysis also showed that a combination of palmitoylation and select structural motifs at the hinge region is needed to enhance the bioactivity of chimeric peptides. Because ADM, ADM2, and CGRP are among the most potent vasodilators (e.g., CGRP is ∼1000 times more potent than acetylcholine and substance P) and protectors of vascular barrier integrity [14,16,50,51,52,53], the gel-forming analogs may represent viable therapeutic candidates for treating a variety of endothelial dysfunction-associated diseases (e.g., RHTN, preeclampsia, and hypertensive acute heart failure).

**Figure 9:**
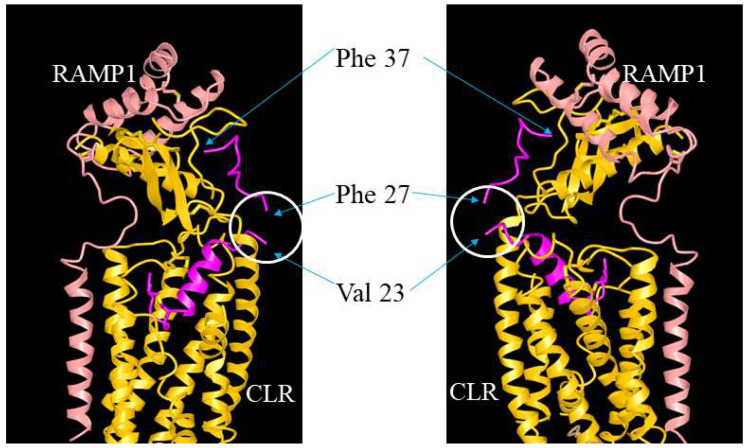
**Illustration of the putative position of the hinge region in CGRP/ADM family peptides and analogs.** The interaction of ADM analogs and CLR/RAMP receptors could be analogous to that between CGRP and CLR/RAMP1 (RCSB protein data bank [PDB] structure 6E3Y). The structure view of CGRP-CLR/RAMP1 complex 6E3Y is depicted from two different angles, and it includes CGRP (red), RAMP1 (pink), and CLR (yellow) components. The unresolved structure at the hinge region is indicated by a white circle. Residues Val23 and Phe27, which are neighboring residues of the hinge region (Lys24-Asn26), and the C-terminal Phe37 of CGRP are indicated by arrows.

ADM, ADM2, and CGRP interact with CLR/RAMP receptors via a two-domain model. The C-terminal binding region binds the receptor ectodomains while the N-terminal activation domain activates the receptor complex [54,55]. In addition, the receptor-binding C-terminus appears to determine the selectivity of these peptides toward the three CLR/RAMP receptors [55]. On the other hand, residues in the hinge region of these peptides may help maintain the C-terminus structure [54,56,57,58,59]. The hypothesis is supported by a structural analysis of CGRP-CLR/RAMP1 complex [55]. CGRP was shown to form extensive interactions with both CLR and RAMP1 with 61.5% of the peptide surface buried, and the CGRP’s N-terminus (Ala1-Val23) tightly interacts with the receptor core [55,60,61] (Figure 9). However, the structure at the hinge region was not clearly resolved, perhaps due to high molecular mobility [55]. Our study of truncated analogs corroborated the idea that the hinge region of these peptides, which corresponds to the junctional region of the chimeric peptides, may play a limited role in the regulation of receptor activation. Although it is not clear how the deletion of 3 residues at the hinge region affects the overall peptide structure, our data indicate that an N-terminal palmitoylation modification may render the truncated analogs to retain an “active conformation” as efficiently as the full-length analogs even in the absence of a normal hinge region.

Lipidation has been used to improve the pharmacokinetics of peptides such as glucagon-like peptide-1 (GLP-1) and glucagon analogs by enabling them to be bound by albumin and other serum proteins, therefore reducing elimination by kidneys and degradation by serum proteases [62]. Earlier studies have shown that lipidated CGRP, CGRP antagonist, and ADM have protracted pharmacokinetic properties [63,64,65]. Unlike earlier studies, we showed that palmitoylation significantly enhances the receptor-activation activities of select ADM and chimeric analogs. Although it is not clear how palmitoylation modification enhances the bioactivity of these analogs, earlier studies of benzoylated and lipidated analogs of CGRP antagonist CGRP8-37 have shown that lipidation may facilitate the association of peptides with the cell membrane and increase the local concentration of peptides within the vicinity of the receptor [65,66,67]. Because a key ligand-binding site in CLR/RAMP receptors is a hydrophobic patch extending from the base of CLR loop 4 to loop 3 [55,66], the palmitoylated analogs may have better interactions with the hydrophobic binding motifs, therefore gaining the enhanced bioactivity. In addition, palmitoylation may provide a better mimic of the membrane environment that a ligand normally encounters when it interacts with a cell surface receptor, therefore improving the conformational dynamics of ligand-receptor interactions [68,69,70,71]. However, an improper combination of ADM/ADM2 sequence in a chimeric analog may drastically disrupt the ligand-receptor interaction even though a facilitating palmitoyl moiety is present.

ADM and ADM2 have organ-protective effects in animals with heart failure, myocardial infarction, stroke, resistant hypertension, pulmonary arterial hypertension, preeclampsia, secondary lymphedema, or diabetic ulcer [19,72,73,74,75,76,77,78,79,80,81,82,83,84,85,86,87,88,89,90,91,92,93,94,95]. These peptides may prevent organ injuries by improving endothelial cell functions and vascular barrier integrity and reducing oxidative stress as well as by improving cardiac output and renal glomerular filtration [6,72,73,74,83,96,97,98,99,100,101]. To improve endothelial functions, ADM could act on the endothelial nitric oxide synthase (eNOS) and inflammatory signaling pathways. The endothelium is a cell layer lining the entire circulatory system, from the heart to the capillaries. It is essential for the regulation of vascular tone, blood flow, thrombosis and thrombolysis, platelet aggregation, and growth of vessels [102]. Endothelial dysfunction is a type of nonobstructive artery disease. It occurs when there is not enough nitric oxide (NO) in vessels, and it can increase endothelium-derived contracting factors, reduce NO production, and disrupt endothelial barrier function, leading to vasoconstriction, inflammation, atherosclerosis, thrombosis, vascular stiffness, and porous vessel walls, which expose tissues to damaging lipoproteins and other harmful substances [103,104,105]. In addition, endothelial dysfunction represents an early biomarker for the development of heart attack and stroke. Studies of ADM and NO signaling have shown that ADM and ADM2 protect vasculatures by stimulating NO production and the eNOS signaling pathway [106,107]. Chronic ADM infusion partly inhibited the increase of blood pressure in association with the restoration of renal neuronal nitric oxide synthases (nNOS) and medullary eNOS expression in hypertensive rats [108]. ADM infusion also increased the NOS coupling and bioavailability in hypertensive rats [109]. In the myocardial ischemia model, ADM deficiency increased the mortality rate whereas exogenous ADM limited infarct development in mice, perhaps by augmenting the phosphorylation of eNOS and Akt proteins [110]. Importantly, the effect of ADM on capillary growth and blood flow recovery in an ischemic model was shown to be abrogated in eNOS-deficient mice, suggesting that a large part of ADM’s regenerative effect in response to ischemia could be mediated through the activation of eNOS pathway [111]. In addition, ADM has been shown to reduce the expression of endothelial dysfunction biomarkers such as intercellular adhesion molecule-1 (ICAM-1) and vascular adhesion molecule-1 (VCAM-1), and inflammatory factors in lymphatic endothelium and VEGF-stimulated human umbilical vein endothelial cells [112,113]. ADM also inhibited angiotensin II- and cold damage-induced up-regulation of ICAM-1, VCAM-1, and other proinflammatory factors in aortic endothelial cells and liver sinusoidal endothelial cells, respectively [114,115]. Similarly, ADM2 exerted vascular and renal protection by inhibiting oxidative stress pathways and ICAM-1 expression in hypertensive rats [116]. Nonetheless, the exact role of ADM on the expression of cell surface endothelial dysfunction biomarkers remains to be investigated. ADM has been shown to induce the expression of VCAM-1 and ICAM-1 on human umbilical vein endothelial cells and was associated with an increase in VCAM-1 and ICAM-1 expression in type 2 diabetes patients [117,118]. Given the importance of eNOS and these cell surface biomarkers in mediating ADM signaling, future studies are needed to determine whether the palmitoylated analogs have a more potent effect on the regulation of NO pathways and the expression of cell surface biomarkers such as VCAM-1 and ICAM-1 as well as other cardiovascular risk factors such as IL-1, IL-6, and tumor necrosis factor-α when compared to wild-type peptides in vivo [119].

In clinical studies, ADM was shown to improve hemodynamics and renal functions in patients with heart failure or pulmonary hypertension [72,76,85,86,87,101] and decrease pulmonary vascular resistance and arterial pressure in patients with pulmonary hypertension [83,87,120]. However, the development of therapeutic ADM peptides was largely bogged down in the last decade because these peptides have short half-lives (i.e., ~20 min) and may induce compensated tachycardia at pharmacological doses [72,73,74,75,76,83,101]. Although the use of lipidated analogs has led to the FDA approval of peptide drugs such as liraglutide and semaglutide [62], they still require frequent injections. On the other hand, studies of self-assemble peptide nanostructures have led to the FDA approval of a self-assembled somatostatin receptor agonist, lanreotide Autogel (25% *w*/*w*; Somatuline Autogel), as a monthly injection for the treatment of acromegaly and neuroendocrine tumors (NETs) [43,44,45,46]. This slow-release approach avoids the need for a complex encapsulation process associated with nanoparticles and has a 100% loading capacity because the only gel-forming molecule in the gel is the peptide therapeutics itself [121]. Because gel-forming ADM analogs not only can be slowly released but also have enhanced receptor-activation activity, they may have the potential to become viable drug candidates for the treatment of severe hypertensive disorders such as RHTN and preeclampsia in an acute or long-term regimen. This idea is further supported by the observation that Analog C-6 gel solution has minimal effect on the heart rate while potently reducing hypertension in SHR rats.

Among the various endothelial dysfunction-associated diseases, we suspect that the gel-forming analogs could be particularly suitable for the treatment of RHTN and preeclampsia because these serious hypertensive disorders are associated with high mortality and severe morbidity and there is a lack of effective treatment. RHTN was defined as a persistent elevation of blood pressure above goal despite concurrent use of 3 antihypertensive agents, each of a unique class with a diuretic included, and with all drugs at the target dose [122]. It has been estimated that 10–15% of the hypertensive population in developed countries met the strict definition of RHTN [123,124,125,126]). RHTN is an important risk factor for cardiovascular and renal events, including stroke, myocardial infarction, heart failure, chronic and end-stage kidney disease as well as cardiovascular mortality [40]. Although antihypertensive treatment options have increased from just three classes in 1970 to over 11 classes now [127,128,129,130,131,132,133,134,135,136,137,138,139,140], physicians are with difficulties achieving controlled blood pressure in these patients. As a result, RHTN patients have a significantly elevated risk of all-cause mortality [122,123,141,142,143,144]. The situation could be related to the fact that most existing antihypertensive drugs act by blocking (1) the renin-angiotensin-aldosterone axis, (2) the sympathetic nervous activity, and (3) the endothelin signaling pathway. There is a lack of therapeutics that can “actively” improve endothelial function and vascular barrier integrity. As such, RHTN is becoming an unfolding healthcare crisis. The gel-forming ADM analogs may make it possible to activate the critical but undrugged endothelium-protective CLR/RAMP signaling in a lasting manner. In addition, these analogs may provide a novel strategy to prevent and treat hypertensive acute heart failure because ADM has been shown to ameliorate heart failure symptoms in various heart failure models and patients.

Because the ability of peptides to form nanostructured gels is governed by multiple forces, including hydrogen bonds, hydrophobic interactions, and π-π aromatic interactions among side chains of the amino acids as well as the concentration of the peptide and adjuvants [47,48,49], future studies of these analog gels with different adjuvants and peptide levels may reveal whether the analog gel formulation can be further improved to provide an even longer period of residence time, therefore reducing the need for frequent dosing.

In addition to cardio- and vessel-protective effects, ADM has potent angiogenic properties. Studies of rodent hindlimb ischemia and lymphedema models have shown that ADM is a therapeutic candidate for improving vasculogenesis and lymphangiogenesis in patients with local trauma or breast cancer-associated lymphedema [18,26,145,146]. Because select gel-forming analogs have significantly enhanced stimulatory effects on HLME cell proliferation and survival as well as the possibility of localized stimulation, they could have improved effects on angiogenesis not attainable with wild-type peptides even at a high dose. As such, these gel-forming analogs may also represent promising candidates for localized treatment of traumatic wounds (i.e., to the tissues surrounding wounds), secondary lymphedema (i.e., inside the lymph nodes), or myocardial infarction (i.e., inside the infarcted region) while avoiding potential systemic adverse effects. However, it is important to caution that whether the analog gel has a balanced efficacy and safety profile remains to be investigated. Although we did not observe a significant increase in heart rate when the blood pressure is reduced by 45–70 mmHg at selected time points, it is conceivable that reflexive tachycardia may be induced at some point after drug administration. Therefore, future studies of the efficacy/safety profiles of these analogs are needed to determine their translational potential for different diseases.

Furthermore, it is important to note that three distinct binding-protein approaches have been taken to extend the half-life of ADM in vivo. First, a combination of ADM and an adrenomedullin-binding protein (AMBP-1) has been shown to reduce organ ischemia/reperfusion injury, presumably by stabilizing the exogenous ADM in circulation [147,148,149]. In the second approach, a humanized ADM-binding antibody (Adrecizumab, HAM8101) was shown to increase plasma ADM levels and reduce systemic inflammation and endotoxin-induced flu-like symptoms in sepsis models [38,150,151,152]. In the third approach, IgG-linked ADM was shown to have a half-life of >2 days; however, the IgG-linked ADM molecules appeared to have drastically reduced receptor-activation activities when compared with wild-type ADM [153]. Although the first two approaches have the potential to treat sepsis or ischemia/reperfusion injury, they may not be suitable for the treatment of hypertensive disorders because, at least, the Adrecizumab was shown to have little effect on hemodynamics. Because the ADM analog gel formulation presumably could provide a larger reservoir of ligands with enhanced bioactivity in vivo when compared with these binding-protein approaches, the analog gel may also be useful for the treatment of sepsis and organ ischemia/reperfusion injury.

## 4. Materials and Methods

### 4.1. Materials

Wild-type and chimeric peptides (>95% purity) were synthesized using the solid-phase peptide synthesis method and obtained from Genscript Inc. (Piscataway, NJ, USA) and Lifetein LLC (Hillsborough, NJ, USA). The identity of purified products was confirmed by MS spectrometry. The mini-PEG moiety used in peptide modification has a molecular weight of 385.42 g/mol and a structure of 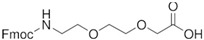. Control reagents included human β-CGRP, human ADM1-52, human ADM14-52, and human ADM2.

### 4.2. Receptor-Activation Assays for CLR/RAMP1, 2, and 3

The bioactivity of peptide analogs was studied using CLR/RAMP1, CLR/RAMP2, and CLR/RAMP3 assays by DiscoveRx (Fremont, CA, USA) in cells that stably express human CLR/RAMP1 (1321N1 cells), CLR/RAMP2 (CHO-K1 cells), or CLR/RAMP3 (CHO-K1 cells). The dose-dependent stimulatory effect of agonists was studied in duplicate, at 10 different concentrations. Determination of the half-maximal effective concentration (EC_50_) was performed using a 10-point dose-response curves. The starting concentration was 1.0 μM, and it was serially diluted 3-fold, in DMSO. Human β-CGRP was used as a positive control in the CLR/RAMP1 assay, and wild-type ADM was used as the control in CLR/RAMP2 and 3 assays.

The agonistic activity was described as EC_50_, and the maximum activity was presented as the percentage of positive control for a given receptor.

### 4.3. Assay of CLR/RAMP1 and 3 Signaling

To assay CLR/RAMP1 and 3 activity, cAMP Hunter cell lines with a select receptor were expanded from freezer stocks [154]. Cells were seeded in a total volume of 20 μL into 384-well microplates, and the activity was determined using the DiscoverX HitHunter cAMP XS + assay. Before the assay, media was aspirated from cells and replaced with 15 μL 2:1 HBSS/10 mM Hepes: cAMP XS + Ab reagent. Intermediate dilution of samples was performed to generate 4X sample in assay buffer, and 5 μL of the 4X sample was added to cells and incubated at 37°C. Vehicle concentration was 1%.

After compound incubation, assay signal was generated by incubating with 20 μL cAMP XS + ED/CL lysis cocktail for 1 h, followed by incubation with 20 μL cAMP XS + EA reagent for 3 h. The chemiluminescent signal was read with a PerkinElmer instrument. The compound activity was analyzed using a CBIS data analysis suite (ChemInnovation, San Diego, CA, USA). The percentage activity was calculated using the following formula: % Activity = 100% × (mean RLU of test sample − mean RLU of vehicle control)/(mean Max control ligand − mean RLU of vehicle control).

### 4.4. Assay of CLR/RAMP2 Receptor Signaling

The CLR/RAMP2 signaling was assayed using a CLR/RAMP2 PathHunter β-Arrestin assay [155]. In this assay, the CLR was fused in frame with a small enzyme donor fragment ProLink™ (PK). The receptor was co-expressed with a fusion protein of β-arrestin and an N-terminal deletion mutant of β-galactosidase (i.e., enzyme acceptor). Activation of the CLR/RAMP2 receptor stimulated the binding of β-arrestin to the PK-tagged receptor and led to an increase in enzyme activities which can then be measured with the PathHunter detection reagents. PathHunter cell lines were seeded in a total volume of 20 μL into 384-well microplates and incubated at 37 °C prior to the assay. Intermediate dilution of samples was performed to generate 5X samples in the assay buffer. A 5 μL aliquot of 5X sample was added to cells and incubated at 37 °C for 90 min. Vehicle concentration was 1%. The assay signal was generated by adding an aliquot of (50% *v*/*v*) of PathHunter detection reagent cocktail, followed by a 1-h incubation at room temperature.

### 4.5. Visual and Microrheology Viscosity Assays of Gel-Forming Capability

We initially determined the gel-forming capability of peptides qualitatively based on a visual examination of the gel solution using a tube-tapping method. Once we have a rough estimate of the gel-forming capability, we quantitatively determine the viscosity of select peptides using a Rheosense viscometer (Rheosense Inc.; http://www.rheosense.com, accessed on 1 December 2019) [49]. In the visual assay, aliquots of peptides were dissolved in an aqueous solution (i.e., de-ionized water, saline, or 5% glucose solution). The peptide that dissolves instantly and stays as a clear solution without obvious macroscopic change in viscosity at 20 min after mixing was considered soluble. If macroscopic particles of peptides remained in the solution for 20 min after mixing, the peptide was considered insoluble. If the peptide solution exhibited a high viscosity at 20 min after mixing, and the solution conformation only change slowly when the vial is tilted 90° and tapped with a finger, it was considered a gel-forming peptide. In addition, we quantitatively determined the viscosity of Analog C-6 and control peptides using a viscometer. The Rheosense microrheology viscometer uses a “chip” flow channel to quantify the viscosity of a solution as small as 50 μL. After loading the aqueous solution, the sample was injected through the flow channel with multiple pressure sensors, and the rheological properties were determined based on the standard principles of rheometry [49].

### 4.6. Measurements of the Passage of Peptide Molecules through Centricon Filters

To evaluate whether gel formation reduces the movement of peptide molecules in solution, we used the Centricon^®^ filters (Millipore) to separate samples and the carriers based on their molecular mass. The sample is monomeric peptide, and the carrier is the self-assembled gel. One-milligram aliquots of the peptides were first dissolved as 0.1%, 5%, or 20% solution in 5% glucose. Ten minutes later, the solution was diluted to the 0.1% level, and aliquots of the samples were dispensed into separate Centricon columns (30,000 MW cutoff). The monomeric peptides and gel molecules were separated by centrifugation for 15 min (2000× *g*). The level of peptide in the elutes was determined by specific EIAs (Phoenix Pharmaceuticals Inc., Burlingame, CA, USA).

### 4.7. Culture of Human Lymphatic Microvascular Endothelial Cells

Primary human lymphatic microvascular endothelial cells (HLME cells; Cell Applications, Inc., San Diego, CA, USA) were maintained in a microvascular endothelial cell growth medium supplemented with 10% fetal bovine serum (FBS). To study the effects of receptor agonists on HLME cell proliferation, aliquots of cells were seeded in 96-well plates and treated with different concentrations of testing compounds or controls (30, 100, and 300 nmoles/L; controls included ADM1-52, ADM14-52, and ADM2) in endothelial cell basal medium supplemented with 2% FBS. After 72-h incubation, cell viability was determined with the 3-(4,5-dimethylthiazol-2-yl)-2,5-diphenyltetrazolium bromide (MTT) assay (Promega). To evaluate the effects on endothelial cell survival, HLME cells were treated with different concentrations of the agonist or controls (30, 100, and 300 nmoles/L) and cultured in a basal medium with 0.1% FBS for 72 h. Cell viability was then assayed with the MTT assay.

### 4.8. Animals and Ethics Statement

All experiments were conducted using adult Sprague–Dawley rats or spontaneously hypertensive rats (SHR) that were 10–14 weeks old. Rats were housed in the Cardio-lab LLC (Gaithersburg, MD, USA) animal care facilities. All procedures were approved by the Institutional Animal Care and Use Committees and conducted in accordance with the National Institute of Health Guide for the Care and Use of Laboratory Animals. Animals were euthanized at the end of the experiment.

### 4.9. Analysis of the Release of Analog C-6 from the Gel Solution In Vivo

Single-dose pharmacokinetics of the Analog C-6 gel solution was investigated in male Sprague–Dawley rats. Rats received a gel solution via the subcutaneous route, and blood samples were collected at pre-dose, 8 h, 1, 2, 4, and 8 days via a catheter that had been cannulated to the jugular vein. Plasma was obtained by centrifugation, and the peptide level in samples was determined by specific ADM EIA (Phoenix Pharmaceuticals Inc.).

### 4.10. Measurement of Hemodynamics in SHR Rats

To study the hemodynamics in adult SHR rats, animals were anesthetized with pentobarbital (40 mg/kg, i.p.) before placing a Mikro-tip catheter in the right carotid artery. Animals were placed in dorsal recumbency, and the ventral cervical area was shaved. The skin was swabbed with surgical scrub (iodine and alcohol), and a 2-cm skin incision was made 2 mm to the right of the trachea. The sternocleidomastoid muscle was separated by blunt dissection and mobilized laterally to locate the right carotid artery. After separating the carotid artery from the vagus nerve, two sterile silk sutures were passed beneath the carotid artery. In relation to the heart, the more distal silk suture was tied to occlude blood flowing from the head region. The proximal silk suture was tied loosely around the carotid artery, and a microclamp was placed on the artery to stop blood flow from the heart. A 20G needle was then inserted into the carotid artery between the two ligatures. The needle was retracted, and the advancing cannula was stopped just before the proximal suture. A Mikro-tip catheter (1.0 F pressure catheter) was inserted into the cannula through the loop, and the loop was tightened to stop bleeding. After insertion, the catheter was advanced into the aorta and stopped when an arterial pressure curve appeared. The catheter was pulled out from the incision of the back neck and fixed to the skin with a suture. The incisions were closed with braided surgical sutures, and the animals were monitored until they become awake.

Animals were given buprenorphine 0.1 mg/kg subcutaneously and placed in a clear tube holder. The Mikro-tip catheter was connected to a Powerlab system, and blood pressure and heart rate were recorded. Compounds were administered by subcutaneous injections. Data were collected at 0 h while animals stayed in the holder. Then animals were placed back in their cages, and a jacket was put on the rat to hold the catheter. Hemodynamics was measured again in the cage at predetermined time points by connecting the catheter to the Powerlab system. Animals were euthanized at the end of the experiment.

### 4.11. Measurements of Dermal Blood Flow and Vasodilation in Rat Hindlimbs

After acclimation, adult male Sprague–Dawley rats were anesthetized and stabilized under 2.5% isoflurane, and placed on a heating pad. The amount of blood flow of the hindlimbs was determined by laser Doppler imaging as described earlier [156]. The laser Doppler scan series began with two baseline scans and were followed by two scans each at select time points. Data are reported as percent change from the average baseline scans.

### 4.12. Statistical Analysis

Comparisons among test groups were performed by the ANOVA test and Student’s *t*-test using the Excel Analysis ToolPak package. The post hoc tests included the Tukey-Kramer or z-test analysis. The data were presented as mean ± SEM, and the significance was accepted at *p* < 0.05.

## Figures and Tables

**Table 1:** EC_50_ values of ADM, ADM2, CGRP, and chimeric analogs.

Identity	CLR/RAMP1		CLR/RAMP2		CLR/RAMP3	
	EC_50_ (nM)	Max Activity	EC_50_ (nM)	Max Activity	EC_50_ (nM)	Max Activity
		% of Controls		% of Controls		% of Controls
**ADM positive**			13–26	100	0.4	104
**CGRP positive**	1–1.5	100				
**ADM2**	116	72	70	67		
**ADM14-52**	540	69	9	102		
**ADM1-52**	564	63	12	91	2.2	130
**CGRP**	1.8–2.9	103	>1000			
						
**Analog A-1**	>1000	1	>1000	8		
**Analog A-2**	**0.8**	47	**0.9**	110		
**Analog A-3**	**0.6**	58	**0.9**	130		
**Analog A-4**	**0.8**	42	**1**	116		
**Analog A-5**	**0.4**	51	**0.6**	126		
**Analog A-6**	**0.2**	50	**0.7**	120		
**Analog A-7**	180	61	7	94		
**Analog A-8**	>1000	2	>1000	4		
**Analog A-9**	>1000	33	19	40		
						
**Analog B-1**	**0.3**	54	**0.5**	143	**0.06**	117
**Analog B-2**	3	54	3	94		
**Analog B-3**	**0.3**	62	**0.4**	101		
						
**Analog B-4**	909	53	149	94		
**Analog B-5**	224	58	17	111		
**Analog B-6**	31	95	18	115		
						
**Analog C-1**	**0.1**	50	**0.3**	79		
**Analog C-2**	**1**	78	>1000	77		
**Analog C-3**	**0.5**	89	**0.7**	97		
**Analog C-4**	**0.2**	82	**0.5**	86		
**Analog C-5**	2	99	**1**	86		
						
**Analog C-6**	24	78	2.8	87		
**Analog C-7**	13	70	**0.6**	147	**0.6**	127
**Analog C-8**	619	69	1.1	68		
**Analog C-9**	28	45	3	90		
**Analog C-10**	34	79	49	46		
						
**Analog D-1**	**0.3**	78	**0.2**	77	**0.07**	141
**Analog D-2**	**0.4**	61	**0.3**	67	**0.2**	156
**Analog D-3**	771	53	17	85		
**Analog D-4**	11	67	1.3	82		
**Analog D-5**	43	71	6.4	74		
**Analog D-6**	7.7	56	**0.3**	74	**0.5**	129
**Analog D-7**	4.5	96	**0.7**	77	**0.3**	140
**Analog D-8**	>1000	6	>1000	6		

## Data Availability

All data are presented in the figures and tables of this study.
